# Supplementary material for: Artificial Intelligence in Healthcare: University Students’ Perceptions and Level of Confidence
Source: Healthcare (Basel). 2025 Sep 16;13(18):2312. doi: 10.3390/healthcare13182312 (PMC12470069; doi:10.3390/healthcare13182312)
Supplement: Supplementary file 1 [file healthcare-13-02312-s001.zip › healthcare-3782471-supplementary.pdf]

Supplementary Material S1: Questionnaire

1. Birth year: ____________

2. Sex:

☐ Female

☐ Male

3. What Faculty do you attend? ____

4. What course do you attend? ____

5. Where did you attend High School?

☐ Portugal

☐ Brazil

☐ PALOP

☐ Another European country. Where? ________________________

☐ Another country outside Europe. Where? _____________________

6. Did you attend a Public or Private High School?

☐ Public school

☐ Private school

7. Artificial Intelligence can be defined as:

☐ A) The use of electronic devices to communicate between people.

☐ B) The study and development of systems that simulate the human capacity to learn and solve problems.

☐ C) The creation of electronic devices aimed exclusively at storing data.

☐ D) The development of social networks for interaction between people in different parts of the world.

8. How do you assess your knowledge of Artificial Intelligence (AI)?

1: No knowledge; 2: Superficial knowledge; 3: Basic knowledge; 4: Advanced knowledge; 5: Proficiency

1	2	3	4	5

9. How do you assess your use of AI tools?

1: Never use; 2: Rarely use; 3: Occasional use; 4: Frequent use; 5: Very frequent use

1	2	3	4	5

10. Choose the option that best characterizes you in relation to the following statement: "I am familiar with the use of AI in healthcare."

1: Strongly disagree; 2: Disagree; 3: Neutral; 4: Agree; 5: Strongly agree

1	2	3	4	5

11. Assinale a opção que melhor o caracteriza em relação à seguinte afirmação: "A sociedade portuguesa encontra-se pronta para implementar ferramentas de inteligência artificial no dia-a-dia."

1: Strongly disagree; 2: Disagree; 3: Neutral; 4: Agree; 5: Strongly agree

1	2	3	4	5

12. Assinale a opção que melhor o caracteriza em relação à seguinte afirmação: "A sociedade portuguesa possui literacia digital acerca das tecnologias de informação, incluindo inteligência artificial."

1: Strongly disagree; 2: Disagree; 3: Neutral; 4: Agree; 5: Strongly agree

1	2	3	4	5

**Choose the option that best characterizes you according to the following statements:**	1: Strongly disagree	2: Disagree	3: Neutral	4: Agree	5: Strongly agree
13. AI will reduce the workload on healthcare professionals.					
14. AI will replace healthcare professionals.					
15. AI will be useful in filling the shortage of healthcare professionals.					
16. AI should be part of the training of health professionals.					
17. Healthcare professionals are informed about the use of AI in healthcare.					
18. AI will improve healthcare.					
19. I would like AI to be implemented wherever possible in the healthcare I receive.					
20. Medical opinion must prevail over the opinion of an AI system.					
21. I trust AI more than a doctor's opinion.					

**Choose the option that best characterizes you according to the following statements:**	1: Strongly disagree	2: Disagree	3: Neutral	4: Agree	5: Strongly agree
22. AI will reduce medical errors.					
23. AI will enable more accurate diagnoses.					
24. AI will put patients' health at risk.					
25. AI will personalize my treatments more than a doctor would.					
26. AI will take my preferences into account more than a doctor would.					
27. With AI, doctors will have more time for their patients.					
28. AI is more susceptible to external influence than a doctor.					
29. I'm afraid that doctors will become dependent on AI.					
30. AI in healthcare will bring new ethical challenges.					
31. AI will put medical confidentiality at risk.					
32. AI will put data protection at risk.					

**Choose the option that best characterizes you according to the following statements:**	1: Strongly disagree	2: Disagree	3: Neutral	4: Agree	5: Strongly agree
33. The doctor using the AI is responsible for any errors arising from its use.					
34. The AI programmers are responsible for any errors arising from its use.					
